# 3D chromatin architecture in cancer: mechanisms of dysregulation and emerging therapeutic strategies

**DOI:** 10.1038/s12276-026-01748-6

**Published:** 2026-06-05

**Authors:** Sunyoung Jang, Kyung Hyun Yoo

**Affiliations:** Laboratory of Biomedical Genomics, Department of Biological Sciences, Sookmyung Women’s University, Seoul, 04310 Republic of Korea

**Keywords:** Cancer genomics, Chromatin

## Abstract

The three-dimensional (3D) genome architecture is fundamental to orchestrating gene expression, safeguarding genome integrity and establishing cell-type-specific transcriptional landscapes. In cancer, this spatial genome architecture becomes profoundly disrupted through alterations in DNA regulatory elements, dysregulation of architectural proteins such as CCCTC-binding factor and cohesin, aberrant chromatin looping, and the breakdown or rewiring of topologically associating domains. Such structural perturbations can activate oncogenic enhancers and super-enhancers, promote inappropriate promoter–enhancer communication, and facilitate large-scale chromatin reprogramming that drives malignant cell states. Recent advances in high-resolution genome conformation technologies including Hi-C, HiChIP and single-cell 3D genomics have uncovered widespread spatial reorganization across diverse tumour types, revealing mechanisms of cancer progression that extend far beyond linear mutations. This review synthesizes current insights into how 3D chromatin structure is altered in cancer, elucidates molecular pathways linking structural dysregulation to oncogenesis and evaluates emerging therapeutic strategies targeting 3D genome architecture, from epigenetic modulators to enhancer disruption and phase-separation-based interventions.

## Disrupted 3D Genome Architecture Drives Cancer Progression

Cancer research has long focused on genetic mutations, but recent studies highlight the critical role of 3D genome architecture in cancer progression. This review explores how disruptions in genome topology, such as topologically associating domain boundary alterations and enhancer–promoter loop rewiring, contribute to oncogene activation and tumour heterogeneity. The authors provide a comprehensive overview of the mechanisms by which structural variations and epigenetic changes reshape the 3D genome, driving cancer progression and therapy resistance. Key findings include the identification of enhancer hijacking as a major driver of oncogene activation and the potential of targeting 3D genome architecture as a therapeutic strategy. The review emphasizes the significance of emerging technologies such as Hi-C and CRISPR-based tools in mapping and manipulating 3D genome structures. By targeting these architectural disruptions, future therapies could offer more precise and effective cancer treatments, marking a new frontier in precision oncology.

This summary was initially drafted using artificial intelligence, then revised and fact-checked by the author.

## Introduction

Cancer cells are characterized by profound transcriptional dysregulation and epigenomic reprogramming. Although decades of research have focused primarily on DNA sequence mutations and chromosomal aberrations, it has become increasingly evident that many critical pathogenic alterations in cancer occur at the level of genome topology rather than DNA sequence alone. Disruption of topologically associating domain (TAD) boundaries, rewiring of enhancer–promoter loops, aberrant formation of long-range chromatin interactions, and large-scale reorganization of nuclear compartments have now been observed across a broad spectrum of tumour types. These architectural abnormalities can activate oncogenes through ectopic enhancer hijacking, silence tumour suppressor genes via insulation loss or compartment switching and generate transcriptional instability that fuels tumour heterogeneity and therapy resistance^1,2^.

Importantly, 3D genome alterations often cooperate with classical genetic and epigenetic lesions to drive malignant progression. Structural variations (SVs), including deletions, inversions, translocations and copy number alterations, can reshape the 3D landscape by repositioning regulatory elements across domain boundaries. In parallel, epigenetic remodelling of histone modifications, DNA methylation and chromatin accessibility can create permissive chromatin environments that stabilize aberrant loops and enhancer networks. This convergence of structural and epigenetic mechanisms underscores the concept that cancer is not merely a disease of altered genes but also a disease of altered genome architecture^3^.

Beyond its mechanistic significance, the cancer 3D genome has emerged as an attractive therapeutic target. Unlike fixed genetic mutations, genome architecture is inherently plastic and potentially reversible. Epigenetic drugs that alter chromatin states, inhibitors that disrupt enhancer activity and emerging strategies targeting transcriptional condensates and phase-separated nuclear domains provide new opportunities to therapeutically reprogramme malignant chromatin structures. Furthermore, recent advances in locus-specific epigenome editing raise the possibility of directly restoring disrupted chromatin boundaries or rewired enhancer–promoter contacts in a highly targeted manner^4,5^.

In this review, we distinguish our work from existing literature — which has predominantly focused on the descriptive mapping of the cancer epigenome — by providing a comprehensive translational roadmap from mechanistic dysregulation to actionable clinical interventions. We summarize current knowledge on how 3D chromatin structure is altered in cancer and provide a conceptual framework linking genome topology to malignant transcriptional programmes. We discuss the major structural features of the 3D genome, the mechanisms through which these features become dysregulated during tumorigenesis and the functional consequences of topological rewiring for cancer initiation, progression and therapeutic resistance. Finally, we highlight emerging therapeutic strategies that seek to modulate or restore genome structure, positioning 3D genome targeting as a new frontier in precision cancer medicine, while critically evaluating the translational barriers that must be overcome to realize this clinical potential.

## Hierarchical structure of the genome

**Fig. 1:**
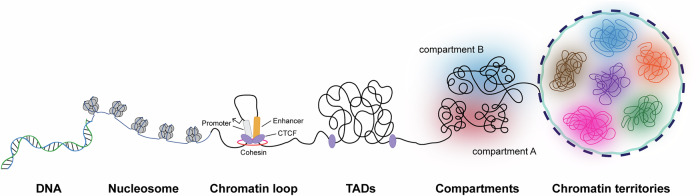
Landscape of 3D genome structure. The human genome is organized into a complex, multi-scale hierarchical architecture that regulates cellular function. DNA is packaged into nucleosomes, which carry various DNA and histone modifications. Chromatin loops are formed through enhancer–promoter interactions and stabilized by architectural proteins, CTCF and cohesin. These loops are organized into topologically associating domains (TADs), which function as self-interacting units. TAD boundaries are maintained by structural proteins, facilitating high-frequency interactions within the domains. On a larger scale, chromatin segregates into A compartments (active) and B compartments (inactive). At the highest scale of organization, each chromosome occupies a distinct spatial region within the nucleus, known as chromatin territory.

The genome is hierarchically organized into multiple nested layers of 3D genome architecture, forming a dynamic regulatory framework that governs gene expression (Fig. 1). At the most basic level, DNA is packaged into nucleosomes and higher-order chromatin fibres, which are then arranged into chromatin loops that physically connect distal enhancers with their target promoters^6,7^. Loop formation is largely driven by cohesin-mediated loop extrusion, with boundary elements frequently defined by CTCF^8,9^. On a larger scale, loops are organized within TADs, which function as insulated regulatory neighbourhoods that restrict enhancer activity to appropriate targets. Beyond TADs, the genome is segregated into transcriptionally active A compartments and inactive B compartments^10,11^. At the highest level, chromosomes occupy discrete nuclear territories and are further organized by nuclear bodies such as transcription factories, nucleoli and Polycomb bodies^12^. Together, these multiscale architectural features establish a regulatory scaffold that coordinates and stabilizes transcriptional programmes in a cell-type- and state-specific manner.

## 3D chromatin dysregulation in cancer

**Fig. 2:**
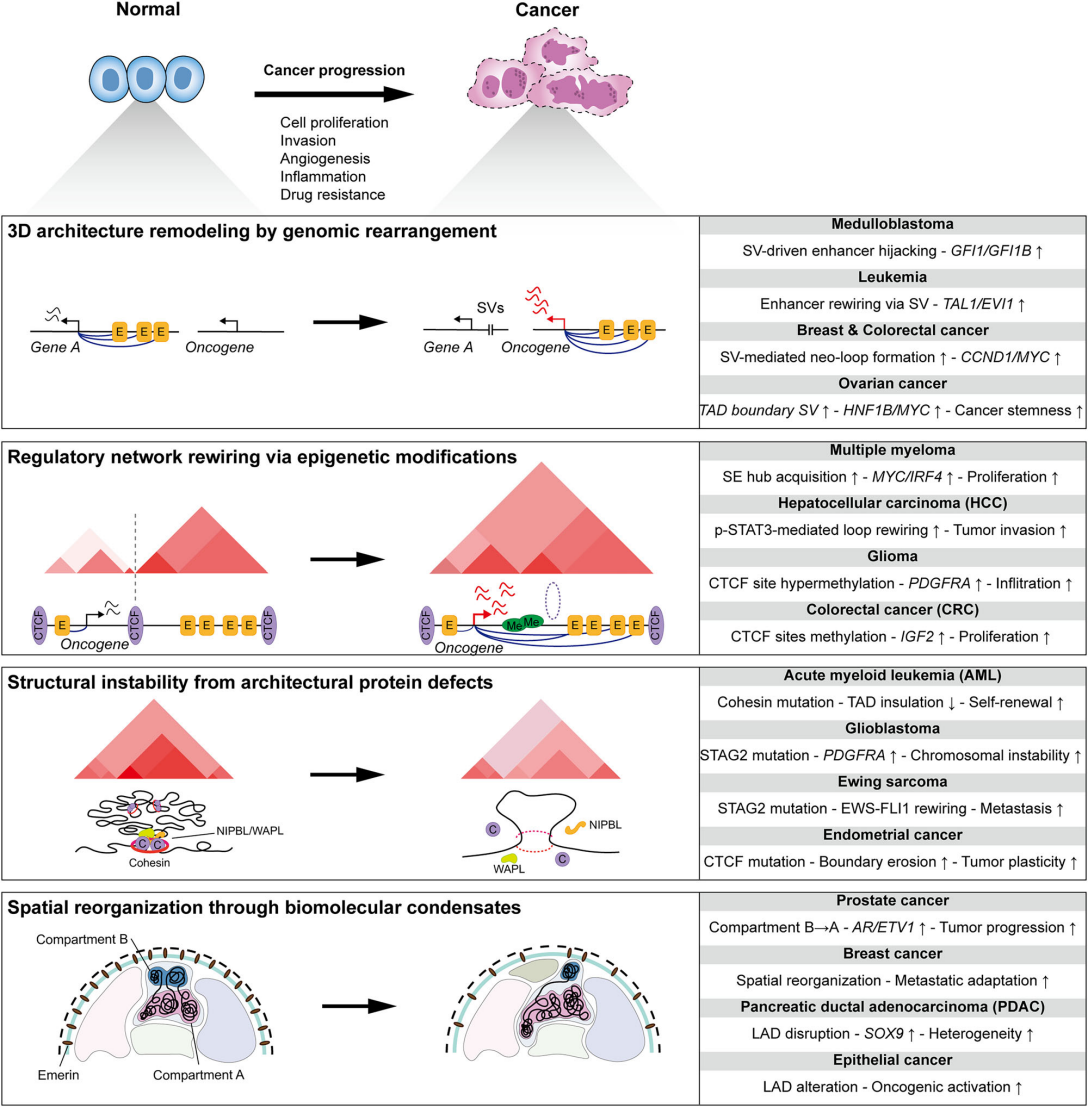
Mechanisms of 3D genome dysregulation during tumorigenesis. Schematic representation of higher-order chromatin structural alterations during tumour progression. Chromatin architecture is disrupted, leading to aberrant gene expression programmes and oncogenic activation. In tumour, large-scale super-enhancers are established through enhanced recruitment of coactivators such as BRD4 and Mediator complex, thereby forming biomolecular condensates that drive aberrant overexpression of key oncogenes. Structural variants (SVs) collapse native chromatin loops, and this “loop rewiring” places distal enhancers to be placed in proximity to unintended oncogenic promoters (*Gene B*) — known as enhancer hijacking — while simultaneously silencing lineage-specific genes (*Gene A*). DNA hypermethylation or mutations at CTCF binding sites lead to the erosion of TAD boundaries, allowing enhancers from neighbouring domains to abnormally activate oncogenes. Loss-of-function mutations in the cohesin complex or its regulators impair the loop extrusions, leading to global weakening of TAD insulation and alterations in the chromatin folding landscape, contributing to genomic instability. Cancer cells exhibit extensive A/B compartment switching, wherein regions harbouring tumour suppressor genes frequently transition from the active A compartment to the repressive B compartment.

Abnormal gene expression patterns can distort cell fate and ultimately lead to cancer. These aberrant transcriptional programmes are thought to be finely controlled by dynamic remodelling of 3D genome architecture. To illustrate how dynamic 3D chromatin remodelling gives rise to aberrant gene expression programmes in cancer, we next highlight representative mechanisms of architectural disruption, including 3D architecture remodelling by genomic rearrangements, regulatory network rewiring via epigenetic modification, structural instability from architectural protein defects and spatial reorganization through biomolecular condensates (Fig. 2).

### 3D architecture remodelling by genomic rearrangements

Structural variants (SVs), including deletions, inversions, tandem duplications and translocations, represent one of the most powerful drivers of 3D chromatin rewiring in cancer. These rearrangements can reposition strong distal enhancers into close spatial proximity with proto-oncogenes, a phenomenon known as enhancer hijacking. Through this mechanism, oncogene activation occurs without any change in the coding sequence itself^13,14^. Classic examples include MYC activation across multiple malignancies, TAL1 activation in T cell acute lymphoblastic leukaemia and GFI1/GFI1B activation in medulloblastoma, where super-enhancers originally controlling unrelated developmental genes become aberrantly linked to oncogenic targets^15^. In adult solid tumours, integrative genome and 3D chromatin analyses have revealed that SV-mediated enhancer hijacking frequently give rises to neo-loop formation, enabling distal enhancers to ectopically engage oncogenes such as MYC, TERT, CCND1 and ERBB2 in colorectal, lung, breast and liver cancers, thereby markedly increasing their transcriptional output^16–18^. In a subset of tumours, this regulatory rewiring is further amplified on extrachromosomal DNA or within complex co-amplified domains, where clusters of enhancers and super-enhancers assemble into high-order 3D regulatory hubs around oncogenes, most prominently MYC, leading to the stabilization of neo-loops and exceptionally high levels of transcription. Hi-C, Capture-C and related chromatin conformation assays have demonstrated that these SV-driven neo-loops create stable enhancer–promoter contacts that persist across cell divisions^19,20^. Furthermore, somatic SVs and boundary-weakening lesions frequently reshape local TAD structures, enabling active enhancers and even super-enhancers to spill over into neighbouring domains and aberrantly activate proto-oncogenes or alternative promoters that are normally insulated in various solid tumours^21,22^. Although SVs can actively rewire 3D chromatin to drive oncogene activation, pre-existing 3D genome features can likewise influence where somatic mutations preferentially accumulate. Emerging evidence suggests that cell-of-origin 3D genome architecture — characterized by distinct mutational signatures between A/B compartments and mechanical strain at TAD boundaries — robustly predicts regional mutation densities. This underscores a model in which the native 3D architecture imprints a non-random mutational template, effectively ‘priming’ the genomic landscape for specific evolutionary trajectories even prior to malignant transformation^23,24^. Ultimately, this synergistic interplay between spatial topology and mutational landscapes underscores that cancer is a disease of both genome sequence and spatial topology.

### Regulatory network rewiring via epigenetic modification

Cancer cells exploit epigenetic plasticity to fundamentally reorganize transcriptional circuitry, a process largely driven by the acquisition and hijacking of super-enhancers (SEs). SEs represent dense clusters of enhancers characterized by an exceptionally high occupancy of transcription factors, Mediator and BRD4. These structures drive the robust expression of genes essential for cell identity and survival while serving as structural scaffolds for 3D genome architecture through long-range interactions and phase-separated condensates^25,26^. Across diverse malignancies — including neuroblastoma, medulloblastoma and multiple myeloma — cancer cells frequently acquire tumour-specific SEs that amplify key oncogenes such as *MYC, MYCN* and *IRF4*, thereby stabilizing malignant transcriptional states^27,28^. 3D chromatin mapping reveals that these cancer-associated SEs often function as highly connected interaction hubs. Within these hubs, a single, hierarchically dominant “hub enhancer” can engage multiple promoters within a TAD or sub-TAD, anchoring complex regulatory networks^29,30^. For instance, in MYC-driven tumours, BRD4-directed SEs organize such multi-promoter networks, whereas in multiple myeloma an IRF4-centred SE hub sustains a feed-forward loop that couples IRF4 and MYC expression to reinforce the oncogenic programme^31,32^. Super-enhancer acquisition and rewiring have been reported across tumours, including colorectal, cervical, lung and breast cancers^33–36^. Tumour-acquired variant super-enhancers frequently converge on key oncogenes such as MYC, VEGFA and super-enhancer-associated long non-coding RNAs, thereby driving proliferation, epithelial mesenchymal transition and organ-specific metastatic programmes^37^. In addition to new SE acquisition, the remodelling of existing regulatory elements through oncogenic signalling and histone modifications further drives 3D structural rewiring. In hepatocellular carcinoma, sustained activation of phosphorylated STAT3 (p-STAT3) triggers a mechanism where p-STAT3-occupied enhancers aberrantly rewire chromatin loops to activate proto-oncogenes within frequently interacting regions. This loop rewiring stabilizes enhancer–promoter interactions that drive the coordinated expression of invasion- and angiogenesis-related genes, contributing to hepatocellular carcinoma aggressiveness. Importantly, these p-STAT3-mediated neo-loops can persist even after STAT3 inhibition, underscoring their critical role in therapeutic resistance^38^. Furthermore, epigenetic alterations at architectural anchors contribute to topological decay. Specifically, DNA hypermethylation at CTCF-binding motifs can reduce insulator strength and weaken TAD boundaries. This is exemplified by the hypermethylation-mediated loss of CTCF binding at the *PDGFRA* locus in *IDH*-mutant gliomas and at the *IGF2/H19* locus, which facilitates abnormal enhancer–promoter communication and subsequent oncogenic activation^39,40^. Such epigenetic-driven rewiring effectively rewrites the regulatory logic of the genome. By converting lineage-restricted or context-specific enhancers into tumour-specific enhancers into potent oncogenic drivers, cancer cells integrate these elements into aberrant 3D regulatory hubs that reinforce and sustain malignant transcriptional programmes.

### Structural instability from architectural protein defects

The integrity of 3D genome architecture relies on architectural proteins that maintain TAD boundaries. Normally, TAD boundaries are anchored by convergent CTCF binding sites and governed by cohesin-mediated loop extrusion, creating insulated neighbourhoods that constrain enhancer–promoter communication within each domain^41^. In cancer, the collapse of these insulation barriers facilitates “enhancer spillover”, allowing potent regulatory elements to gain unauthorized access to neighbouring proto-oncogenes. A well-characterized example is boundary deletion near the TAL1 and LMO2 loci in T cell acute lymphoblastic leukaemia, which allows enhancer elements from adjacent domains to activate these oncogenes, and rearrangements in medulloblastoma that rewire TAD architecture around GFI1/GFI1B and other drivers^42^. In addition to *cis*-acting structural rearrangements, *trans*-acting mutations in chromatin architectural regulators contribute directly to 3D genome instability^43^. Recurrent mutations in the cohesin complex — specifically affecting components such as *STAG2, SMC1A, SMC3* and *RAD21* — are pervasive across acute myeloid leukaemia, Ewing sarcoma and glioblastoma^44–46^. These cancer-associated variants diminish TAD insulation strength and alter loop extrusion capacity, leading to a profound reshaping of long-range chromatin contacts and the dysregulation of cell-identity genes^47–49^. Beyond the core cohesion ring, dysregulation of cohesion loader and unloader such as NIPBL and WAPL further perturbs loop length, residence time and chromatin mobility, resulting in localized transcriptional imbalances^50^. Likewise, mutations in CTCF itself and somatic mutational hotspots at persistent CTCF binding sites, together with DNA methylation-mediated disruption of CTCF-binding sites compromise insulator function, leading to boundary erosion^51^. Although many of these architectural mutations are insufficient to initiate transformation on their own, they establish a permissive 3D chromatin environment that amplifies the impact of oncogenic enhancers and transcription factors, thereby accelerating the global transcriptional reprogramming necessary for tumour progression. Consequently, these architectural defects reduce the barrier for de novo enhancer–promoter interactions. By maintaining a state of persistent topological instability, mutations in the cohesion complex and CTCF facilitate the formation of aberrant regulatory contacts, thereby providing the transcriptional plasticity required for tumour evolution and therapy resistance.

### Spatial reorganization through biomolecular condensates

On the megabase scale, cancer cells frequently display a large-scale spatial reorganization of the nuclear landscape, characterized by large-scale switching between A (active) and B (inactive) compartments. These transitions serve as a hallmark of higher-order genome remodelling during tumorigenesis and metastasis. Transitions from A to B compartments are often associated with stable gene repression, particularly of tumour suppressor genes or differentiation programmes, whereas B to A switching brings previously silenced regions into transcriptionally permissive nuclear environments^52,53^. These shifts correlate strongly with alterations in histone modifications, chromatin accessibility and long-range interaction frequencies^54,55^. For instance, prostate cancer models exhibit coordinated B to A shifts at androgen receptor (AR)-responsive loci, alongside A to B transitions at regions losing structural features^56,57^. Moreover, metastatic breast and prostate cancer cells exhibit compartment patterns that partially mimic their target organs, suggesting that organotropism-associated architectural reorganization facilitates metastatic adaptation^58^. In parallel with compartment reorganization, cancer cells exhibit disruption of lamina-associated domains (LADs), which normally tether repressive chromatin to the nuclear periphery via interactions with A- and B-type lamins. Loss or remodelling of LAD structure has been documented in pancreatic ductal adenocarcinoma, melanoma, invasive breast and prostate cancers, and is characterized by detachments of heterochromatic domains from the nuclear lamina and their subsequent relocalization toward the nuclear interior. This spatial shift is often accompanied by widespread reductions in peripheral H3K9me2 and DNA methylation and relocalization of these regions towards the nuclear interior^59,60^. Altered expression and mislocalization of nuclear envelope components, including lamin A/B, lamin B1 and emerin, further destabilize nuclear architecture, increase nuclear deformability and are associated with enhanced invasiveness in breast and prostate cancer models, highlighting nuclear positioning as a modulatable component of tumour progression^61,62^. Integrating these large-scale shifts, emerging evidence suggests that biomolecular condensates formed through liquid–liquid phase separation (LLPS) act as the underlying physical mechanism for these spatial reorganizations. High-density transcriptional hubs, such as those formed by super-enhancers, create phase-separated environments that stabilize A-compartment identities and facilitate long-range interaction hubs. Conversely, the dissolution of heterochromatic condensates can drive the erosion of B-compartments and LAD detachment^63,64^. Together, compartment switching, LAD disruption, and phase-separated condensate formation reflect a global reprogramming that reinforces oncogenic transcriptional states and provides the spatial plasticity necessary for cancer cells to adapt to diverse metastatic environments.

## Therapeutic strategies targeting 3D chromatin structure in cancer

**Fig. 3:**
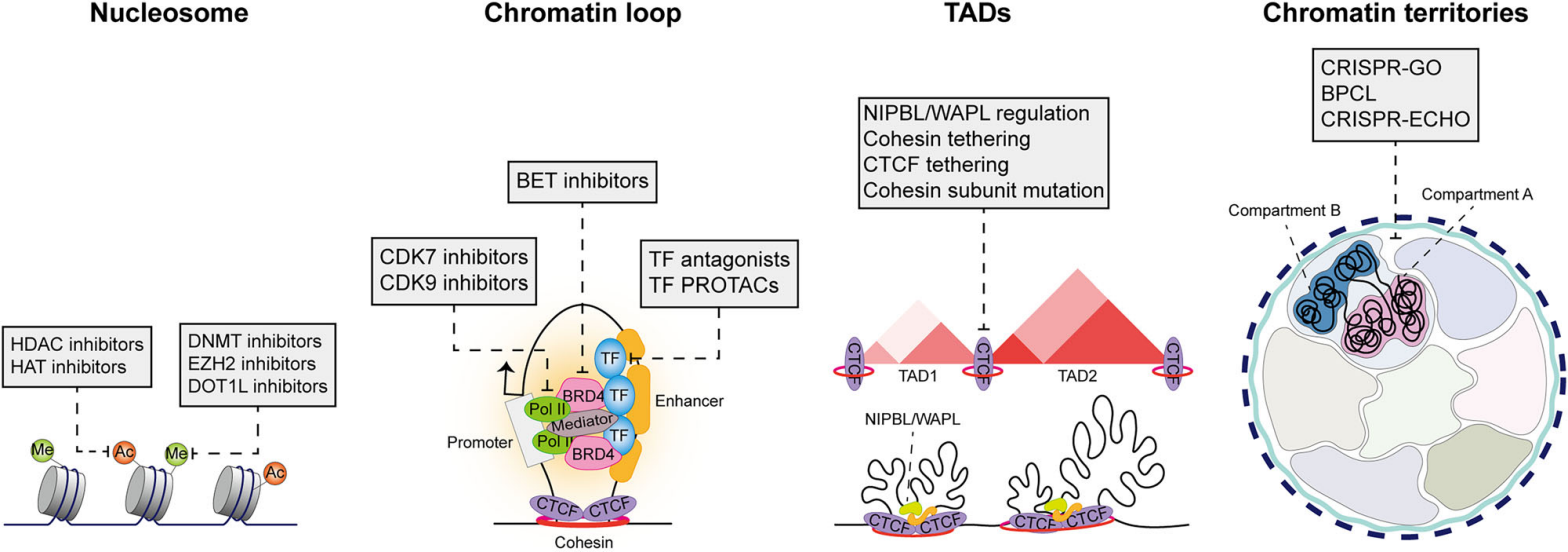
Strategies targeting 3D genome architecture in cancer. Overview of hierarchical genome architecture and corresponding epigenetic and structural intervention strategies. The diagram illustrates the hierarchical layers of genome organization and the pharmacological or molecular tools available to modulate each level. Small molecule inhibitors target histone modifications to reverse aberrant chromatin states. HDAC and HAT inhibitors modulate acetylation levels, whereas DNMT, EZH2 and DOT1L inhibitors reset histone methylation patterns to restore the expression of silenced tumour suppressor genes. Targeting the protein assemblies at oncogenic enhancers prevents the formation of abnormal transcriptional hubs. BET inhibitors and TF PROTACs disrupt super-enhancer-driven “addiction”, whereas CDK7/9 inhibitors block the transcriptional machinery recruited to these 3D chromatin structures. Structural integrity is restored by targeting the cohesin-CTCF axis. Synthetic biology approaches that artificially tether cohesin or CTCF to eroded boundaries reinstate insulation and prevent ectopic enhancer–promoter interactions. Large-scale nuclear architecture can be modulated using programmable CRISPR-based tools, enabling the deliberate relocation of genomic loci between active (A) and inactive (B) compartments. This precision engineering approach offers a strategy to reposition oncogenes into repressive environments near the nuclear periphery.

Although direct manipulation of genome folding remains an emerging therapeutic frontier, multiple drug classes already influence 3D chromatin architecture either directly or indirectly by reshaping epigenetic states, enhancer activity, and nuclear architecture. Because genome topology is inherently dynamic and reversible, these strategies offer unique opportunities to reprogramme malignant transcriptional ecosystems rather than targeting single oncogenes. We have summarized these therapeutic strategies into four major categories: epigenetic remodelling of 3D chromatin architecture, disruption of super-enhancer hubs and biomolecular condensates, targeting loop extrusion dynamics and architectural proteins, and precision engineering of locus-specific 3D topology (Fig. 3 and Table 1).

**Table 1:** Overview of current and emerging drugs targeting 3D genome architecture.

Category	Mechanism	Target class	Drug/compound	Cancer types (examples)	Clinical status	Refs.
Epigenetic remodelling of 3D chromatin architecture	Relaxes 3D chromatin structure and remodels enhancer accessibility	HDAC inhibitor	Vorinostat (SAHA)	CTCL, Solid tumours	FDA approved	^65^
	Relaxes chromatin compaction and alters loop accessibility		Romidepsin	T cell lymphoma	FDA approved	^66,67^
	Relaxes chromatin compaction and alters enhancer accessibility	weak HDAC inhibitor	Valproic acid	Glioma, leukaemia	FDA approved	^66,67^
	Restores CTCF binding and TAD insulation via DNA demethylation	DNMT inhibitor	Azacitidine	AML, MDS	FDA approved	^68^
	Reactivates silenced boundary elements and tumour suppressors	DNMT inhibitor	Decitabine	AML, solid tumours	FDA approved	^68,69^
	Disrupts Polycomb-repressive domains and long-range silencing loops	EZH2 inhibitor	Tazemetostat	Follicular lymphoma, epithelioid sarcoma	FDA approved	^70,71^
Disruption of super-enhancer hubs and biomolecular condensates	Disrupts BRD4-driven super-enhancer hubs	BET inhibitor	JQ1	MYC-driven cancers	Preclinical	^72,73^
	Suppresses super-enhancer-dependent oncogene transcription		OTX015 (MK-8628)	Leukaemia, glioblastoma	Phase I/II	^78^
	Induces BRD4 degradation and structural collapse of enhancer hubs	BET-PROTAC	ARV-771	Breast cancer, leukaemia	Preclinical	^77^
	Collapses BRD4-based enhancer clusters	BET degrader	dBET6	AML	Preclinical	^76,77^
	Blocks RNA Pol II activation at super-enhancers	CDK7 inhibitor	Samuraciclib (CT7001)	Breast cancer, solid tumours	Phase I/II	^79^
	Disrupts transcriptional condensates		THZ1	MYCN-amplified cancers	Preclinical	^27,79^
	Disrupts nucleolar phase condensates	RNA Pol I inducer	CX-5461	Haematological malignancies	Phase I/II	^82^
Targeting loop extrusion dynamics and architectural proteins	Blocks interaction with PDS5B and inhibits cohesin loading and loop extrusion	Cohesin-PDS5B inhibitor	HRO761	STAG2-mutant cancers	Phase I	^86^
Precision engineering of locus-specific 3D topology	Enables targeted enhancer silencing or structural boundary restoration	Programmable chromatin modulation	CRISPR-dCas9 epigenetic editors	Research-stage	Preclinical	^89,90^

### Epigenetic remodelling of 3D chromatin architecture

Epigenetic modifications fundamentally control chromatin compaction, loop formation, and nuclear compartmentalization, making epigenetic regulators powerful indirect modulators of 3D genome architecture. Histone deacetylase (HDAC) inhibitors such as vorinostat (SAHA) and romidepsin relax chromatin structure, increase global accessibility and rewire enhancer–promoter communication networks, often reversing the pathological chromatin compaction that characterizes many malignancies^65–67^. DNA methyltransferase (DNMT) inhibitors, including decitabine and azacitidine, can restore CTCF occupancy at previously hypermethylated binding sites, thereby partially rescuing disrupted TAD boundaries and insulator function, which ultimately drives robust anti-tumour response-resistant tumour growth — including the induction of apoptosis and suppression of therapy-resistant tumour growth — by comprehensively reprogramming oncogenic transcription networks^68,69^. Histone methyltransferase inhibitors, most notably EZH2 inhibitors such as tazemetostat, reshape Polycomb-mediated repressive domains and alter long-range interactions between silent chromatin compartments. By dismantling these densely packed repressive hubs, these agents successfully reactivate silenced tumour suppressor genes and differentiation programmes, thereby driving profound anti-proliferative effects and tumour regression^70,71^. In parallel, BET bromodomain inhibitors such as JQ1 and OTX015 disrupt BRD4-dependent enhancer clustering and super-enhancer addiction, collapsing oncogenic transcriptional hubs^72,73^. Collectively, these agents regulate chromatin interactions as a downstream consequence of modifying epigenetic landscapes, effectively rewiring the 3D regulatory scaffold of cancer cells.

### Disruption of super-enhancer hubs and biomolecular condensates

Super-enhancers represent particularly attractive 3D therapeutic targets owing to their high spatial clustering, dense coactivator occupancy and outsized transcriptional influence on oncogene expression. Tumour cells are frequently “addicted” to super-enhancer-driven transcription of key oncogenes such as MYC, MYCN, and IRF4 (refs. ^74,75^). Although classical BET inhibitors such as OTX015 demonstrate baseline efficacy in downregulating oncogenes and stemness markers, BRD4-directed degraders (e.g. MZ-1, VHL-JQ1 and ARV-771) induce more profound collapse of enhancer hubs by eliminating rather than merely inhibiting BRD4. This irreversible structural collapse completely dismantles oncogenic transcriptional networks, thereby overcoming therapeutic resistance and driving potent synergistic anti-tumour efficacy across diverse malignancies, including multiple myeloma and triple-negative breast cancer^76–78^. CDK7 inhibitors such as samuraciclib suppress transcriptional elongation at super-enhancers by blocking RNA polymerase II activation, a targeted mechanism that has now been clinically validated to disrupt oncogenic transcription and yield substantial clinical benefit in advanced, drug-resistant breast cancer^79^. Furthermore, growing evidence indicates that transcription factors, coactivators and chromatin regulators form biomolecular condensates via LLPS at super-enhancers and transcription factories^80,81^. Key components such as BRD4, MED1, RNA polymerase II and lineage-defining transcription factors assemble into dynamic transcriptional droplets that concentrate regulatory activity. Pharmacological disruption of LLPS has therefore emerged as a novel therapeutic concept. Agents inducing nucleolar stress, such as CX-5461, further illustrate how perturbation of phase-separated nuclear bodies can selectively impair cancer-cell survival by driving the formation of repressive liquid droplets that forcefully exclude transcriptional machinery^82^. Similarly, additional Mediator-disrupting strategies are now under development, aimed at dismantling super-enhancer condensates at their structural core^83^. Although still in its infancy, phase separation-targeted therapy offers the prospect of dismantling complex oncogenic enhancer–promoter hubs without globally suppressing transcription^84^. Ultimately, these agents collectively function by shrinking enhancer clusters and dampening oncogene transcription at the level of 3D regulatory architecture.

### Targeting loop extrusion dynamics and architectural proteins

Targeting the core architectural machinery that governs genome folding is conceptually attractive but remains at an early developmental stage. Modulation of cohesin loading and unloading dynamics through regulatory factors such as NIPBL and WAPL offers a potential means of tuning loop extrusion length and residence time. Recent studies highlight that perturbing these dynamics drastically alters higher-order chromatin compaction and rewires enhancer–promoter communication, thereby exposing selective vulnerabilities in cancer cells and dismantling essential oncogenic programmes^85,86^. Preclinical efforts are also exploring peptide-based inhibitors that interfere with CTCF–DNA binding, aiming to transiently weaken insulator strength and alter domain structure. By deliberately dislodging these fundamental architectural anchors, such interventions induce the targeted collapse of TAD boundaries and massively rewire constrained enhancer–promoter networks, directly challenging the structural dependencies of tumours^87,88^. Although these strategies are not yet clinically mature, they establish genome topology itself as a druggable regulatory layer.

### Precision engineering of locus-specific 3D topology

Beyond broad architectural modulation, recent synthetic approaches have enabled targeted interventions at defined genomic loci. Engineered dCas9 fusions tethered to cohesin or looping factors have demonstrated programmable formation or disruption of specific chromatin loops. By deliberately forcing de novo enhancer–promoter contacts or insulating oncogenic drivers, these targeted systems can precisely switch gene expression states on or off. This provides a compelling proof-of-concept that complex architectural defects may be therapeutically repairable at specific disease-associated regions without globally perturbing the 3D genome^89,90^. These cutting-edge strategies represent a shift toward highly precise 3D genome editing, offering a direct mechanism for correcting enhancer–promoter miswiring or restoring disrupted insulation in cancer.

## Technologies enabling 3D genome-based cancer therapeutics

Recent technological advances have rapidly expanded the translational potential of 3D genome research in cancer by enabling high-resolution mapping, functional perturbation, and real-time visualization of chromatin architecture. Chromosome conformation capture-based technologies such as Hi-C, Micro-C and HiChIP now allow systematic identification of cancer-specific chromatin loops, TAD boundary disruptions, and enhancer–promoter rewiring at kilobase- to nucleosome-level resolution. These approaches have revealed tumour-specific interaction hubs, super-enhancer-driven regulatory circuits, and neo-loops created by structural variations, thereby uncovering architectural vulnerabilities that are not detectable from linear genome analysis alone^91,92^.

The emergence of single-cell 3D genome technologies, including single-cell Hi-C and multimodal single-cell approaches integrating chromatin conformation with transcriptomic and epigenomic profiling, has further transformed the field by enabling direct interrogation of intratumoural architectural heterogeneity. These methods reveal that cancer cells within the same tumour often display highly diverse 3D genome configurations, which correlate with transcriptional states, metastatic potential and drug resistance. Such insights provide a foundation for understanding how architectural plasticity contributes to tumour evolution and therapeutic failure^93,94^.

Functional interrogation of 3D genome architecture has been enabled by CRISPR-based perturbation platforms. CRISPR interference or activation (CRISPRi/a) allows targeted modulation of enhancer activity, while direct deletion of enhancers, CTCF boundaries or loop anchors uncovers causal relationships between chromatin topology and gene regulation. More recently, targeted restoration of TAD boundaries and synthetic loop engineering using dCas9-based tethering systems have demonstrated that pathological chromatin architecture can be experimentally rewired in a programmable manner. These tools provide essential validation frameworks for translating 3D regulatory concepts into therapeutic strategies^95,96^.

In parallel, live-cell 3D genome imaging technologies, including real-time fluorescence-based chromatin labelling, have enabled direct visualization of chromatin dynamics, loop formation and compartment switching in living cancer cells. These approaches reveal that genome architecture is highly dynamic rather than static, responding rapidly to oncogenic signalling, metabolic stress and drug exposure^97,98^. Finally, the integration of artificial intelligence and deep-learning-based predictors has enabled in silico reconstruction of chromatin contact maps from DNA sequence or epigenetic features, facilitating large-scale prediction of cancer-specific architectural vulnerabilities^99–101^. Together, these technologies form an integrated experimental–computational pipeline for identifying and targeting druggable features of the 3D cancer genome.

## Challenges and future perspectives

Despite rapid progress, several fundamental challenges must be overcome before 3D genome-based cancer therapeutics can be fully realized. To successfully translate these preclinical and early clinical breakthroughs into precision oncology, the field must address critical barriers related to functional validation, context specificity, system-level safety, and multi-omics data integration.

### Distinguishing functional drivers from passenger noise

A primary barrier lies in distinguishing driver architectural alterations from pervasive passenger noise generated by genome instability. Although thousands of chromatin interaction changes are detected in individual tumours, only a subset directly contributes to oncogenic transcriptional programmes. Establishing robust criteria to define functional driver loops, boundaries and compartments remains a major unmet need.

### Context specificity and 3D genome atlases

A second challenge involves understanding the cell-type and context specificity of genome folding, which complicates the extrapolation of architectural mechanisms across different tumour types. The same structural variation or epigenetic lesion may have distinct architectural and transcriptional consequences depending on lineage identity, differentiation state and microenvironmental cues. To address this, the field is shifting from cell-line models to generating 3D atlases directly from primary tumours. Importantly, these tumour-derived connectomes reveal that although large-scale compartments remain relatively conserved, fine-scale enhancer–promoter loops are highly lineage and tumour specific. This topological specificity establishes patient-derived 3D features as powerful tools for precise cancer classification and tailored therapeutic strategies.

### Target specificity and delivery challenges

Therapeutic manipulation of genome architecture also raises concerns regarding off-target and systems-level effects. Because architectural proteins and epigenetic regulators act globally, unintended disruption of essential 3D genome features in normal tissues could lead to genotoxicity or transcriptional instability. The development of locus-specific and conditionally activated architecture-modifying strategies will therefore be critical for clinical translation. Furthermore, CRISPR-based epigenome- and loop-editing tools have demonstrated proof-of-concept in experimental systems, but their translation into therapeutic modalities will demand further innovation in delivery, specificity and long-term safety.

### Integration with multi-omics and predictive modelling

Another major challenge lies in the integration of 3D genome data with multi-omics profiles including genomics, epigenomics, transcriptomics, proteomics and metabolomics to enable truly personalized cancer medicine. Effective clinical deployment will require predictive models that can translate patient-specific 3D genome configurations into actionable therapeutic decisions.

## Conclusion

Cancer is increasingly recognized as a disease of genome architecture as much as a disease of genome sequence. The pathological disruption of chromatin loops, TAD boundaries, enhancer landscapes and spatial compartments does not merely accompany oncogenesis but actively drives it. Consequently, targeting these structural derangements represents a critical paradigm shift in cancer treatment.

Despite the translational hurdles outlined above, the convergence of high-resolution 3D genome mapping, functional genome engineering, and computational modelling now positions genome topology as a tractable and highly promising therapeutic dimension. As these technologies mature, they will enable a shift from treating the symptoms of transcriptional dysregulation to correcting its structural root causes. Ultimately, 3D genome-guided therapies are poised to profoundly reshape precision oncology by targeting not just the isolated oncogenes but also the intricate, higher-order regulatory infrastructures that sustain malignant cell states.
